# Evaluation of the scabicidal effect of a single dose of fluralaner in a rabbit model of crusted scabies

**DOI:** 10.1007/s00436-023-07945-w

**Published:** 2023-09-01

**Authors:** Mahmoud S. Sharaf, Ahmad A. Othman, Amira E. Abd El Ghaffar, Dareen M. Ali, Mohamed M. Eid

**Affiliations:** 1 Parasitology Department, Faculty of Medicine, Tanta University, Elgeish Street, Tanta, Egypt; 2 Pathology Department, Faculty of Medicine, Tanta University, Tanta, Egypt

**Keywords:** Scabies, *Sacrcoptes scabiei*, Ivermectin, Fluralaner, Treatment

## Abstract

Recently, scabies was included in the WHO roadmap for neglected tropical diseases 2021–2030. Till now, ivermectin is the only available oral drug that is currently approved for treating crusted scabies in humans. Concerns regarding its efficacy and safety have prompted research efforts to find new alternatives. Our study aimed to evaluate the therapeutic effect of a single dose of fluralaner in cases of crusted scabies in comparison with that of repeated weekly high doses of ivermectin. For the *in vitro* study, twenty adult female mites were exposed to 50 μg/ml and 100 μg/ml ivermectin and fluralaner to evaluate their effects on mites' survival. For the *in vivo* study, thirty-five male crossbreed rabbits were divided into 4 groups: group I (non-infected, non-treated), group II (infected, non-treated), group III (infected and treated with ivermectin in a weekly oral dose of 0.4 mg/kg body weight/rabbit for 4 weeks, starting 8 weeks post-infection), and group IV (infected and treated with fluralaner given as a single oral dose of 25 mg/kg body weight/rabbit, starting 8 weeks post-infection). Clinical, parasitological, histopathological, and biochemical assessments were done. Clinical and parasitological assays were accomplished to all infected groups starting from day 0, then on days 2, 4, 6, 8, 10, 12, 14, 21, 28, 35, 42, 49 and 56 post-treatment, while histopathological and biochemical assessments were done at the end of the 8^th^ week post-treatment (day 56). Our results showed that fluralaner exhibited a higher acaricidal effect on adult *Sarcoptes scabiei* var. *cuniculi* when compared with ivermectin applied in the same concentration (50 μg/ml or 100 μg/ml). Concerning the *in vivo* study, both clinical cure and parasitological cure were noted in both treated groups, evidenced by complete absence of all clinical signs of infestation and absence of mites in all skin scrapings. However, the ivermectin-treated group showed incomplete histopathological and biochemical resolution. Interestingly, both clinical cure and negative skin scrapings were noticed earlier in the fluralaner-treated group, with no apparent side effects. Also, no significant differences were noticed in the skin sections and serum biochemical parameters when compared with those of the negative control group. We concluded that fluralaner is a promising scabicidal agent that is recommended to be studied for possible human use, especially in control programs.

**Graphical abstract:**

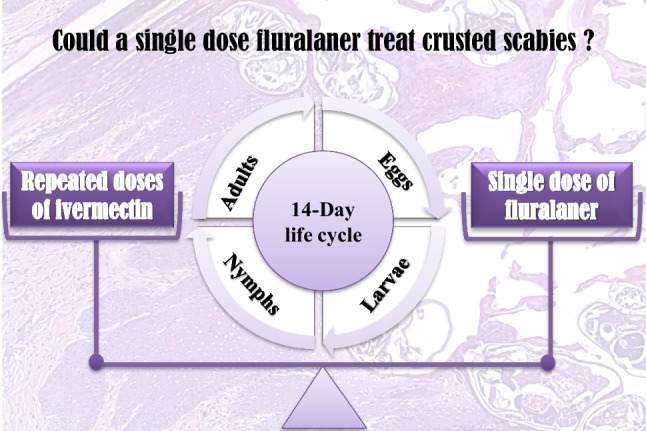

## Introduction

Crusted scabies (CS) is a serious debilitating form of scabies caused by excessive proliferation of *Sarcoptes scabiei* (*S. scabiei*) mites. Its global burden is not precisely known; however, scabies is generally estimated to affect 200–300 million individuals annually worldwide, which is an unacceptably high prevalence for a neglected disease (Leung et al. 2020). Recently, scabies was included in the WHO roadmap for neglected tropical diseases 2021–2030 (WHO 2020).

While scabies could affect people of all ages globally, children and the elderly in low-resource areas are the most vulnerable (El-Moamly 2021). Crusted scabies usually affects immunocompromised individuals (Karimkhani et al. 2017). Generally, infestation with* S. scabiei* is associated with a spectrum of immune responses in the host, resulting in severe itching, inflammation, alopecia, and skin lesions. Crusted scabies is characterized by the development of thick scaly crusts, skin fissuring, and scratching, which cause increased susceptibility to secondary bacterial infections (Næsborg-Nielsen et al. 2022).

Till now, ivermectin (IVM) is the only available oral drug that is currently approved for treating CS in humans, mostly in outbreaks as mass drug administration in endemic areas or poorly compliant patients (Romani et al. 2015). It is a macrocyclic lactone that acts by activating glutamate-gated chloride channels (GluCls) mainly and to a lesser extent GABA-gated chloride channels in invertebrates, like the scabies mites, leading to chloride ions influx, hyperpolarization, and paralysis (Xu et al. 2017).

Since IVM has no ovicidal activity, a single-dose oral IVM is inadequate and multiple doses are required for treating CS or even mild cases to cover the mite life cycle, which is not ideal for mass drug administration (Bernigaud et al. 2020). Additionally, concerns regarding its efficacy (Mounsey et al. 2017) and safety in certain situations (e.g., young children, during pregnancy or breast feeding) have prompted research efforts to discover new alternatives to be used in treatment of scabies (Bernigaud et al. 2016).

Fluralaner (FLR) is a new acaricide of the isoxazoline class that acts by antagonizing GABA-gated chloride channels mainly, and to a lesser extent GluCl-gated chloride channels, blocking the influx of chloride ions, leading to depolarization, excessive neuronal stimulation and death of the mite (Gassel et al. 2014). Since FLR is slowly metabolized in the liver and binds abundantly and tightly to plasma proteins, it has a sustained activity for more than one month (Kilp et al. 2016). Therefore, it is now under consideration as a promising alternative agent for treating CS (Wilkinson et al. 2021). Our study aimed to evaluate the therapeutic effect of a single dose of FLR in cases of CS in comparison with that of repeated weekly high doses of IVM.

## Materials and methods

### Animals

Parasite-free 35 male crossbreed rabbits, two-month-old, weighing 1500-2000 gm were included in our study. Rabbits had no recent history of drug intake. Rabbits were allocated in separate cages in a well-ventilated room at 25 ± 2 ^o^ C under a 12/12-h light/dark cycle, with free access to water and standard food (a commercial pellet diet). Rabbits were allowed to stabilize and acclimatize to the new environment 1 week before induction of infection. All procedures on animals were reviewed and approved by the Research Ethics Committee and Quality Assurance Unit in Tanta University (Approval code: 34838/8/21).

### Parasite

**Fig. 1:**
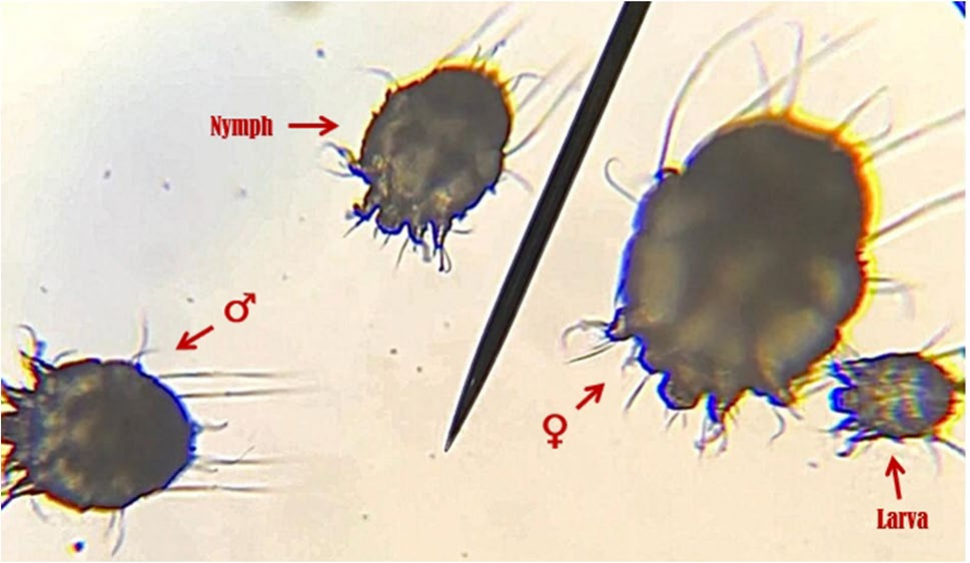
Different stages of *S. scabiei* mites under microscope (×100).

Three naturally infected rabbits, suffering from sarcoptic mange, were employed as a source of *S. scabiei* var *cuniculi* mites for experimental induction of CS. Infestation with scabies mites was confirmed in such rabbits by scrapping the edges of skin lesions with a scalpel blade till capillary bleeding was evident. After placing the collected scales in petri dishes, they were incubated at 30^o^ C for 30 minutes to enhance migration of mites to the surface of the dishes (Bergvall 2005). Finally, a stereoscopic examination of dishes was done to identify the characteristic morphological features of the *S. scabiei* mites (Fig. 1). The mite-infested skin crusts (approximately containing about 600-800 mites) were transferred to the mite-free rabbits' ear canals for induction of infection (Bernigaud et al. 2016). Crusts were applied on pieces of gauze, which were then plastered to the inner surface of the ear of each rabbit to prevent crusts from falling off. Such pieces were removed after 1 hour.

### Drugs

In the IVM-treated group, iverzine® 6 mg oral tablets (UNIPHARMA, Egypt) were given as a weekly oral dose of 0.4 mg/kg body weight/rabbit for 4 weeks, starting 8 weeks after induction of infection (D0) (Kachhawa et al. 2013). In the FLR-treated group, bravectoTM 250 mg chewable tablets (MSD Animal Health, Austria) were given as a single oral dose of 25 mg/kg body weight/rabbit, starting 8 weeks after induction of infection (D0) (Sheinberg et al. 2017). For the *in vitro* assay, both drugs were used in two concentrations, 50 μg/ml and 100 μg/ml, diluted in phosphate-buffered saline (Mounsey et al. 2017).

### *In vitro* assay

Twenty adult female mites were collected from scraped skin lesions in the positive control group of rabbits, exposed to 50 μg/ml and 100 μg/ml IVM and FLR to evaluate their effects on mites' survival. Adults exposed to phosphate-buffered saline without any acaricide were used as a negative control. The count of viable mites in each group was assessed hourly in the first 3 hours post-exposure, then at 24 hours post-exposure. Mortality was considered by absence of any movement when mites were gently touched by a probe as described by Mounsey et al. (2017). Assessment was performed five times for each group and the mean results were taken.

### *In vivo* assay

Our *in vivo* study consisted of 2 phases: the post-infection phase (progression of the infestation to the crusted form) and the post-treatment phase (after drug administration). Each phase lasted 8 weeks. Rabbits were divided into 4 groups: group I (5 non-infected non-treated rabbits, as a negative control); group II (10 infected non-treated rabbits, as a positive control); group III (10 rabbits with CS, treated with IVM); and group IV (10 rabbits with CS, treated with FLR). Clinical and parasitological assays were performed starting from D0, then on days 2, 4, 6, 8, 10, 12, 14, 21, 28, 35, 42, 49 and 56 post-treatment. Histopathological and biochemical assessments were done at the end of the 8^th^ week post-treatment (D56).

#### Clinical evaluation

Rabbits were ranked according to a modified scoring system that was formulated based on the clinical scores used by Jensen et al. (2002), Nong et al. (2013), Casais et al. (2014), Bernigaud et al. (2018) and Hampel et al. (2018). Each animal was individually scored for each parameter, then the total clinical score was calculated. A clinical score of 17 is the maximum total clinical score that one rabbit could be assigned (Table 1). For evaluation of pruritis, rabbits were observed for 15 min to record any sign of pruritis, such as intense chewing, itching, licking, rubbing on a surface, or scratching ears with the posterior legs.

**Table 1:** Parameters used for clinical scoring of treated and untreated rabbits

Clinical findings		Score
1- Extension of crusty lesions [limb lesion score + ear/nose lesion score]		
Limb lesions	No limb lesions observed.	0
	Lesions ≤ 15.5 cm^2^	1
	Lesions 15.6 – 31 cm^2^	2
	Lesions 31.1 – 64 cm^2^	3
	lesions > 64 cm^2^	4
Ear/nose lesions	Early lesions on nose or ear (various focal skin lesions without crusts)	+1
	Focal thin crusty lesions on nose or ear	+2
	Focal thick crusty lesions on nose or ear (involving > 1/2 the ear's margin or covering most of the nose)	+3
	Diffuse thick crusty lesions affecting both ear and nose (all around the ear's margin, covering most of the nose and adjacent parts of the head).	+4
2- Type of lesion		
No skin lesion observed		0
Erythema without other lesions		1
Moist lesions with serous exudation		2
Thin crusts (1-2 mm)		3
Crusts of 3-5 mm thickness, bloody skin lesions.		4
Thick hard crusts (> 5 mm), bloody skin lesions.		5
3- Pruritis		
No pruritis		0
Occasional episodes		1
Frequent episodes + self-trauma (as excoriation, bloody skin lesions).		2
4- Alopecia		
No alopecia (hair regrowth)		0
Areas with alopecia with partial hair (re-) growth		1
Areas with alopecia with no hair (re-) growth		2

#### Parasitological evaluation

Counting viable mites was accomplished during the stereoscopic examination of skin scrapes collected from 2 cm^2^ area from skin lesions in each rabbit, then percentage reduction in viable mite count was calculated. For assessment of the egg hatchability, ten eggs were isolated one by one with a needle from the skin scrapings taken at D0, D2, D4, D6, D8, D10, and D12 post-treatment, placed in a sterile plastic petri dish, incubated at 37°C with 90% relative humidity, and observed in 24 h intervals for 5 days. Eggs were considered dead if they failed to hatch within 5 days (Bernigaud et al. 2020). A total of three replicates were performed for each group, and the mean percentage of hatching rate was taken.

#### Histopathological evaluation

Tissue samples were carefully collected from ear pinna from each rabbit, then samples were preserved in 10% formol saline for histopathological evaluation. Slides were prepared as per standard protocol for staining with hematoxylin and eosin and evaluated microscopically for dermal and epidermal changes by a scoring system modified from scores described by Moats et al. (2016) and Salvadori et al. (2016) (Table 2). The pathologist was blinded to the allocated group.

**Table 2:** Histopathological scoring system

Feature	Measure	Attributed scores			
		0	1	2	3
Crust	Thickness (mm) determined in eight randomly selected microscopic cross sections of skin	No crust	<2.5	2.5-3.5	>3.5
Alopecia	Percent of hair follicles containing hairs in histologic section	Normal	>55%	45-55%	<45%
Hyperkeratosis	× normal width	Normal	2×	3×	>4×
Acanthosis	Cell layers	Normal	3-4 cell layers	5-6 cell layers	>7 cell layers
Mites	Average counts at 40× high-power field (HPF) in eight randomly selected fields	0	1-2	3-6	>6
Epidermal inflammation	Average number of inflammatory cells at 40× HPF in eight randomly selected fields	<5	6-25	26-50	>50
Dermal inflammation	Average number of inflammatory cells at 40× HPF in eight randomly selected fields	<5	6-25	26-50	>50
Total score		0-21			

#### Biochemical evaluation

At D56 post-treatment, blood samples were collected from the marginal ear vein (2 ml from each rabbit) in serum separating vacutainer tubes, allowed to clot for 1 hour at room temperature, then centrifugation was done for 20 minutes at 3,000 rpm. Finally, serum samples were collected in Eppendorf tubes and stored at – 20^o^C till use. Serum levels of C-reactive protein (CRP) (Rabbit CRP ELISA Kit, Cat. No. MBS166122, MyBioSource, San Diego, USA) and malondialdehyde (MDA) (Rabbit MDA ELISA Kit, Cat. No. MBS2602584, MyBioSource, San Diego, USA) were assessed by double-antibody sandwich enzyme-linked immunosorbent assay technique, using available commercial kits according to the manufacturer's instructions.

#### Statistical analysis

Statistical analysis was done using the mean and standard deviation by SPSS V.22. The percentage reduction in mite's count was calculated using the formula (Mpre – Mpost / Mpre) X 100, where M pre represents the mean number of live mites before treatment and M post is the mean number of live mites post-treatment. ANOVA test (f test) was used for comparison among different times in the same group.

## Results

### *In vitro* evaluation

**Fig. 2:**
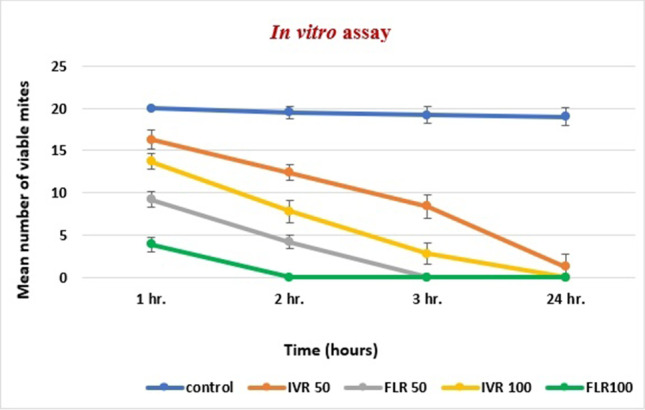
Mean number of viable adult female mites in different replicates when treated with 50 μg/ml and 100 μg/ml ivermectin and fluralaner.

Fluralaner showed a higher acaricidal  effect against *S. scabiei* var. *cuniculi* when compared with IVR applied in the same concentration (50 μg/ml or 100 μg/ml), with a statistically highly significant differences for both concentrations 1 hour, 2 hours and 3 hours post-exposure (*P* value = 0.001***) (Table 3 and Fig. 2).

**Table 3:** The percentage reduction (%) of viable mites treated by 50 μg/ml and 100 μg/ml IVM and FLR

Percent reduction (%)	1 hour	2 hours	3 hours	24 hours
IVR 50	18.5 %	36.4 %	56.2 %	93.1 %
FLR 50	54 %	78.5 %	100 %	100 %
IVR 100	31.5 %	60 %	85.4 %	100 %
FLR 100	80.5 %	100 %	100 %	100 %

### Clinical evaluation

**Fig. 3:**
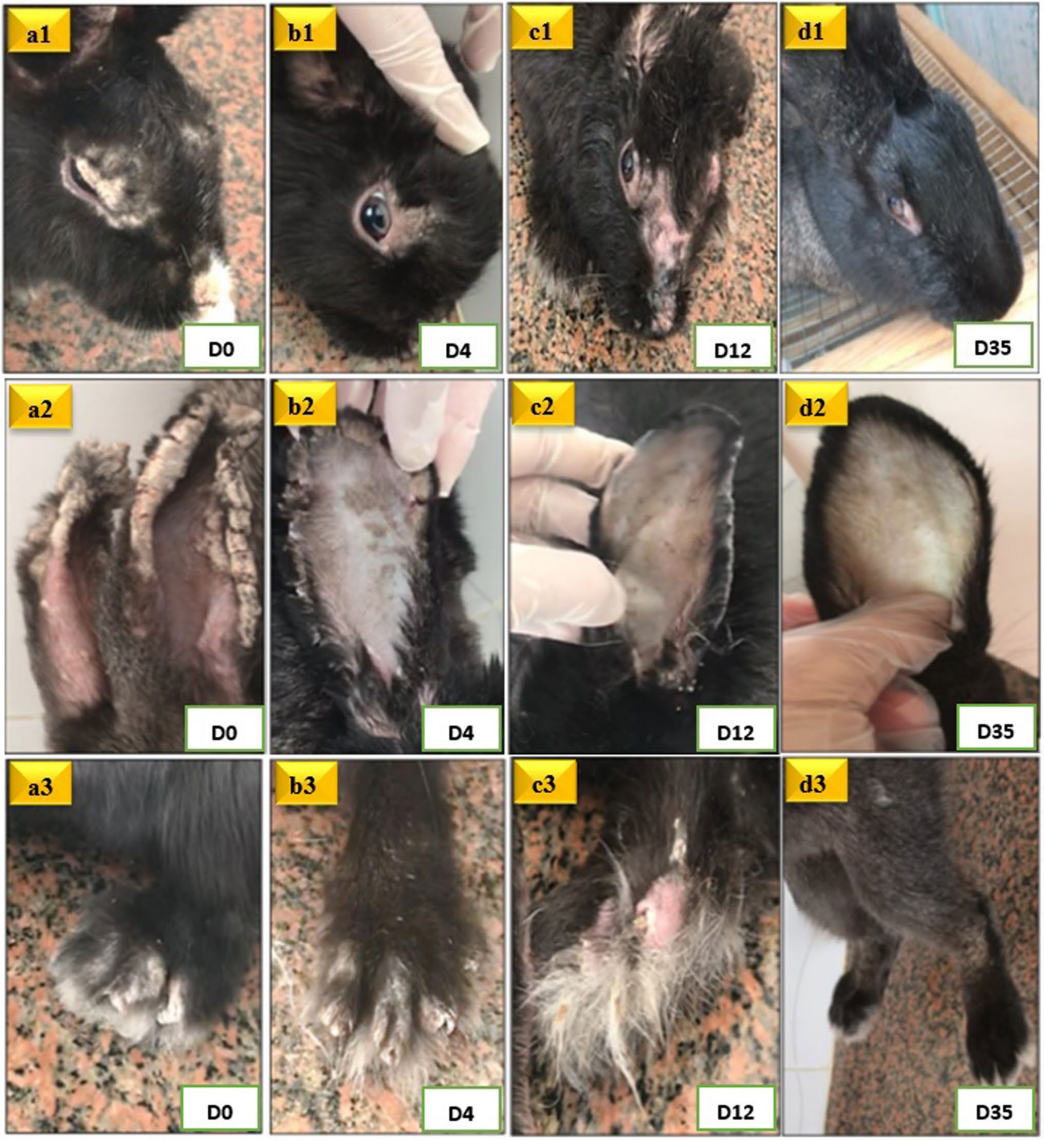
Mangy rabbits treated with IVM. Figures a1, a2 and a3 represent rabbits at D0, with characteristic mangy lesions in face, nose, eyelids (a1), ears (a2), and limbs (a3). Figures b1, b2 and b3 represent rabbits at D4 post-treatment, showing the start of crusts' separation. Figures c1, c2 and c3 represent rabbits at D12 post-treatment, showing complete separation of crusts with a notable skin erythema and alopecia. Figures d1, d2, and d3 represent rabbits at D35 post-treatment, showing complete clinical recovery, evidenced by healing of skin lesions and hair growth.

**Fig. 4:**
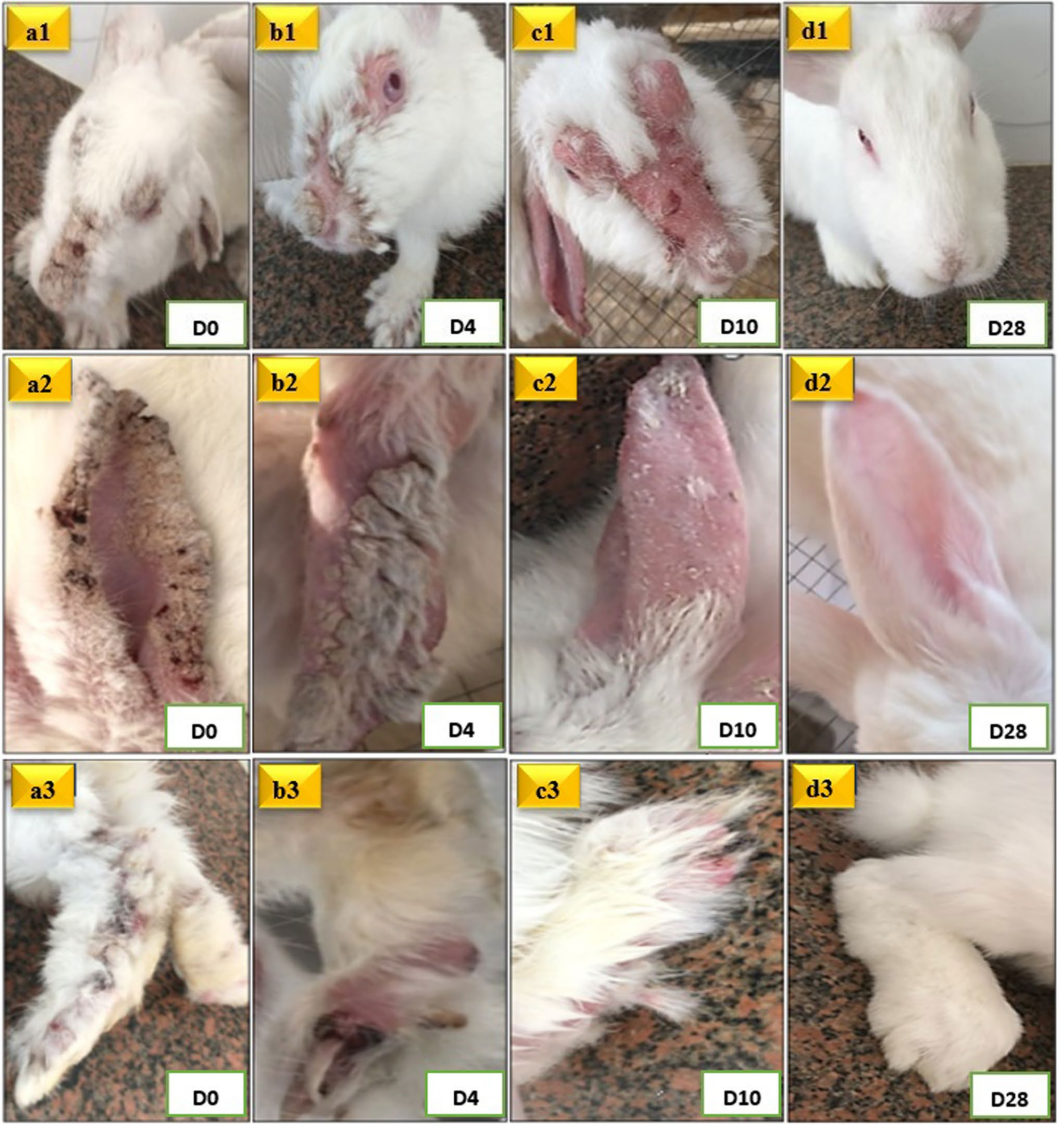
Mangy rabbits treated with FLR. Figures a1, a2 and a3 represent rabbits at D0, with characteristic mangy lesions in face, nose, eyelids (a1), ears (a2), and limbs (a3). Figures b1, b2 and b3 represent rabbits at D4 post-treatment, showing the start of crusts' separation. Figures c1, c2 and c3 represent rabbits at D10 post-treatment, showing complete separation of crusts with a notable skin erythema and alopecia. Figures d1, d2, and d3 represent rabbits at D28 post-treatment, showing complete clinical recovery, evidenced by healing of skin lesions and hair growth.

**Fig. 5:**
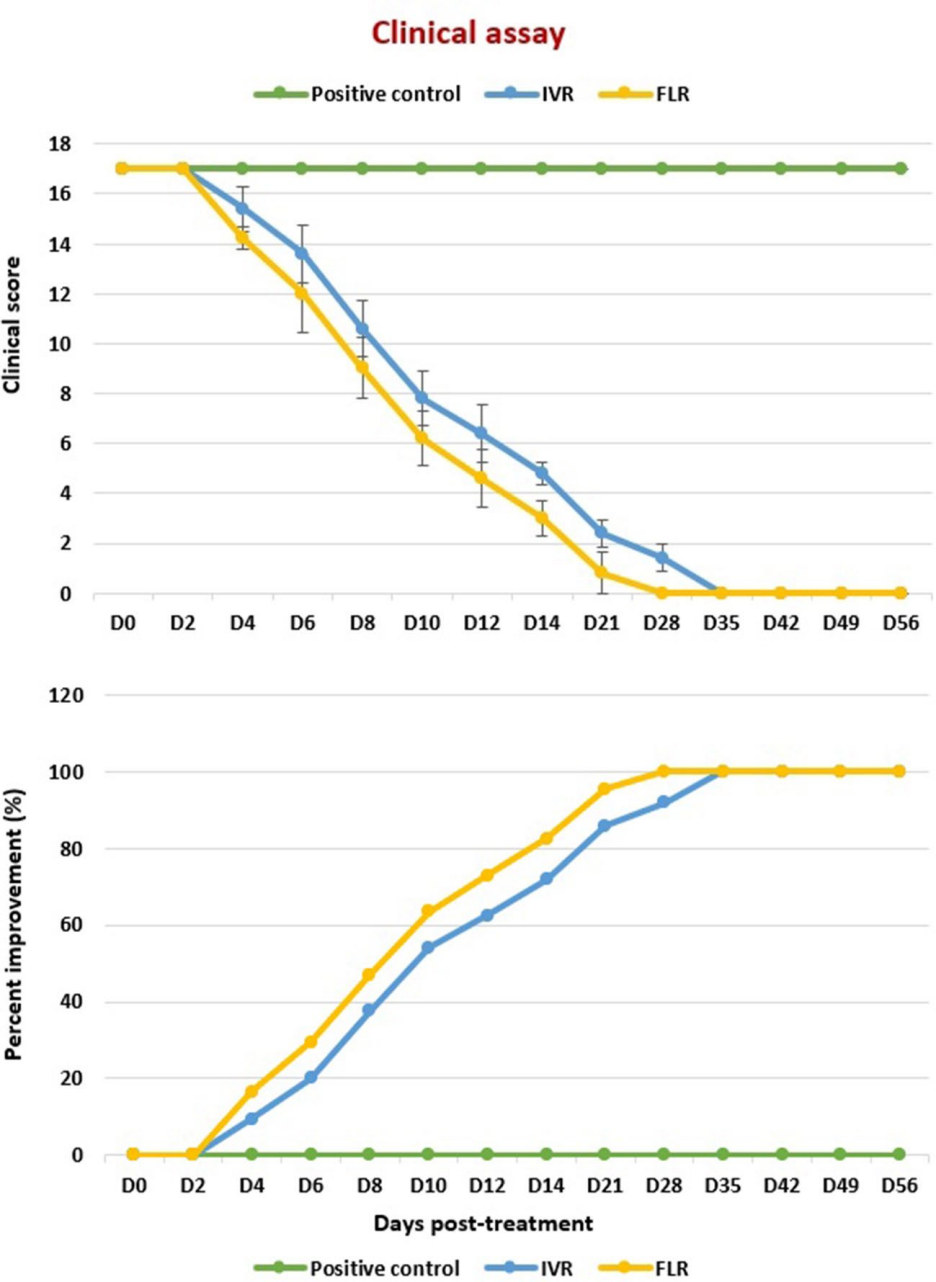
Clinical scores (mean + SD) and percentage clinical improvement of treated and untreated groups.

On D0 (8 weeks post-infection), all infected groups exhibited the characteristic mangy lesions, including marked lichenification, alopecia, hemorrhagic crusts, and fissures, mainly on limbs, ears, face, nose, and eyelids. These lesions were associated with pruritis, intermittent scratching of the affected areas with front paws, emaciation, pale oral and conjunctival mucosae, hypothermia, and enophthalmos (clinical score= 17+0.00) (Fig. 3_a1, a2, a3_, Fig. 4_a1,a2,a3_, and Fig. 5).

In IVM-treated group, a significant progressive clinical improvement in skin lesions and general condition was noticed, starting from D4 post-treatment, compared to clinical status of lesions at D0 in the same rabbits. At D4 post-treatment, falling of crusts started to be evident, with increased frequency of bouts of pruritis (clinical score=15.4+0.89, *P*= 0.01*) (Fig. 3_b1, b2, b3_ and Fig. 5). By D12 post-treatment, no crusts were observed in all rabbits treated with IVM (clinical score= 6.4 + 1.14, *P*= 0.001***), with new hair growth observed starting from D12 post-treatment (Fig. 3_c1, c2, c3_ and Fig. 5). Complete clinical recovery was accomplished at D35 following treatment (clinical score= 0+0.00, *P*= 0.001***) till the end of the experiment (D56 post-treatment) (Fig. 3_d1,d2,d3_ and Fig. 5). Occasional bouts of pruritis were evident in some rabbits till D28 post-treatment.

After a single dose of FLR in the FLR-treated group, a highly significant progressive clinical improvement was noticed, starting from D4 post-treatment with a highly significant progressive reduction in the clinical scores, compared to that at Day 0. At D4 post-treatment, falling of crusts started to be evident (clinical score= 14.2+0.45, *P*= 0.001***), with increased frequency of bouts of pruritis (Fig. 4_b1,b2,b3_ and Fig. 5). By D10 post-treatment, no crusts were observed in all rabbits. Additionally, new hair growth was noticed, and the animals’ behavior was almost normal, although there were still occasional bouts of pruritus (clinical score= 6.2+1.10, *P*= 0.001***) (Fig. 4_c1,c2,c3_ and Fig. 5). The itching disappeared completely starting from D21 post-treatment. Skin lesions showed complete clinical recovery from D28 post-treatment till the end of the experiment (D56 post-treatment) (Fig. 4_d1,d2,d3_ and Fig. 5).

When comparing the clinical scores of rabbits treated with IVM with those treated with of FLR, a more significant clinical improvement was noticed in rabbits treated with FLR than those treated with IVM (*P*= 0.028*, 0.048*, 0.037*, 0.001***, 0.007**, and 0.001*** on D4, D10, D12, D14, D21, and D28). The percentage improvement in rabbits treated with IVM was about 91.76% at D28 post-treatment, while percentage improvement in rabbits treated with FLR was about 100% at D28 post-treatment. However, clinical cure was fulfilled in both groups. Starting from D35 post-treatment to the end of the experiment, no significant difference for all clinical parameters was noted between both treated groups (Fig. 5).

Regarding the adverse effects related to the therapeutic agents used in this experiment, rabbits treated with IVM exhibited diarrhea and decreased food intake, especially during the first few days following each dose of IVM. In contrast, no apparent adverse effects related to FLR treatment were observed in any rabbit during the 8-week post-treatment observation period. No deaths occurred in any treated group throughout the experiment.

### Parasitological evaluation

Table 4 summarizes data regarding the mean counts of viable mites found in the three 2 cm^2^ scrapings in each treated group during the period of the treatment and follow-up. Microscopic examination of skin scrapings obtained from infested rabbits at D0 revealed a large number of eggs, developing and adult mites per microscopic field. Negative skin scrapings were noticed starting from D14 post-treatment in the IVM- treated group and from D10 post-treatment in the FLR-treated group, with a highly significant differences between both groups at D2, D4, D6, D8, D10, D12, and D14 (*P*= 0.001***).

**Table 4:** Mean values (+S.D.) of the viable mite count in treated mangy rabbits

	IVM					FLR					t. test	*P*. value
	Mean + S.D.			% Reduction	*P* value with D0	Mean + S.D.			% Reduction	*P* value with D0		
D0	501.4	±	13.78	-	-	515.6	±	26.03	-	-	1.078	0.312^a^
D2	465.8	±	16.45	7.10	0.016*	427.2	±	8.76	17.14	0.001***	4.631	0.002**
D4	396.2	±	15.01	20.98	0.001***	331.8	±	25.52	35.64	0.001***	4.864	0.001***
D6	182	±	37.40	63.70	0.001***	131.2	±	18.19	74.55	0.001***	2.731	0.026*
D8	136	±	32.32	72.88	0.001***	19.2	±	6.22	96.27	0.001***	7.935	0.001***
D10	46.4	±	12.10	90.75	0.001***	0	±	0.00	100	0.001***	8.578	0.001***
D12	16.2	±	9.78	96.77	0.001***	0	±	0.00	100	0.001***	3.703	0.006**
D14	0	±	0.00	100	0.001***	0	±	0.00	100	0.001***	-	-
D21	0	±	0.00	100	0.001***	0	±	0.00	100	0.001***	-	-
D28	0	±	0.00	100	0.001***	0	±	0.00	100	0.001***	-	-
D35	0	±	0.00	100	0.001***	0	±	0.00	100	0.001***	-	-
D42	0	±	0.00	100	0.001***	0	±	0.00	100	0.001***	-	-
D49	0	±	0.00	100	0.001***	0	±	0.00	100	0.001***	-	-
D56	0	±	0.00	100	0.001***	0	±	0.00	100	0.001***	-	-

- Superscripts indicates statistical significance: ^(a)^ insignificant: *P* > 0.05, ^(*)^ Significant: *P* < 0.05, ^(**)^ very significant: *P*<0.01, ^(***)^ highly significant: *P* <0.001

- *n*= 10 in both groups

**Fig. 6:**
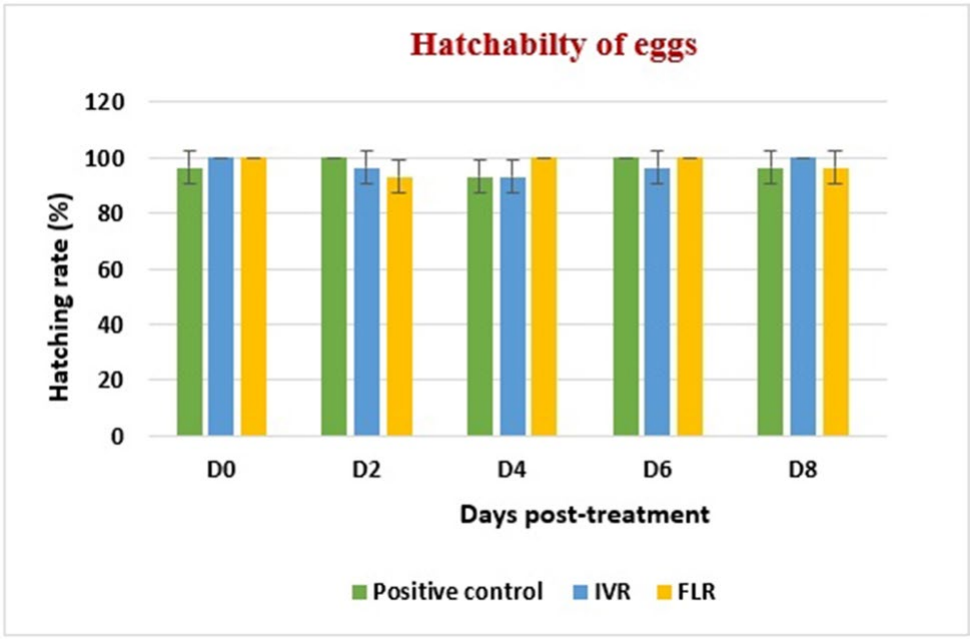
Hatching rate percentage (mean + SD).

Regarding egg hatchability, the vast majority of eggs retrieved from both positive control group and treated groups hatched as shown in (Fig. 6). Starting from D12 post-treatment in the IVM-treated group and D10 in the FLR-treated group, no eggs were found in skin scrapings, whereas almost all the eggs from the positive control group hatched.

### Histopathological evaluation

**Fig. 7:**
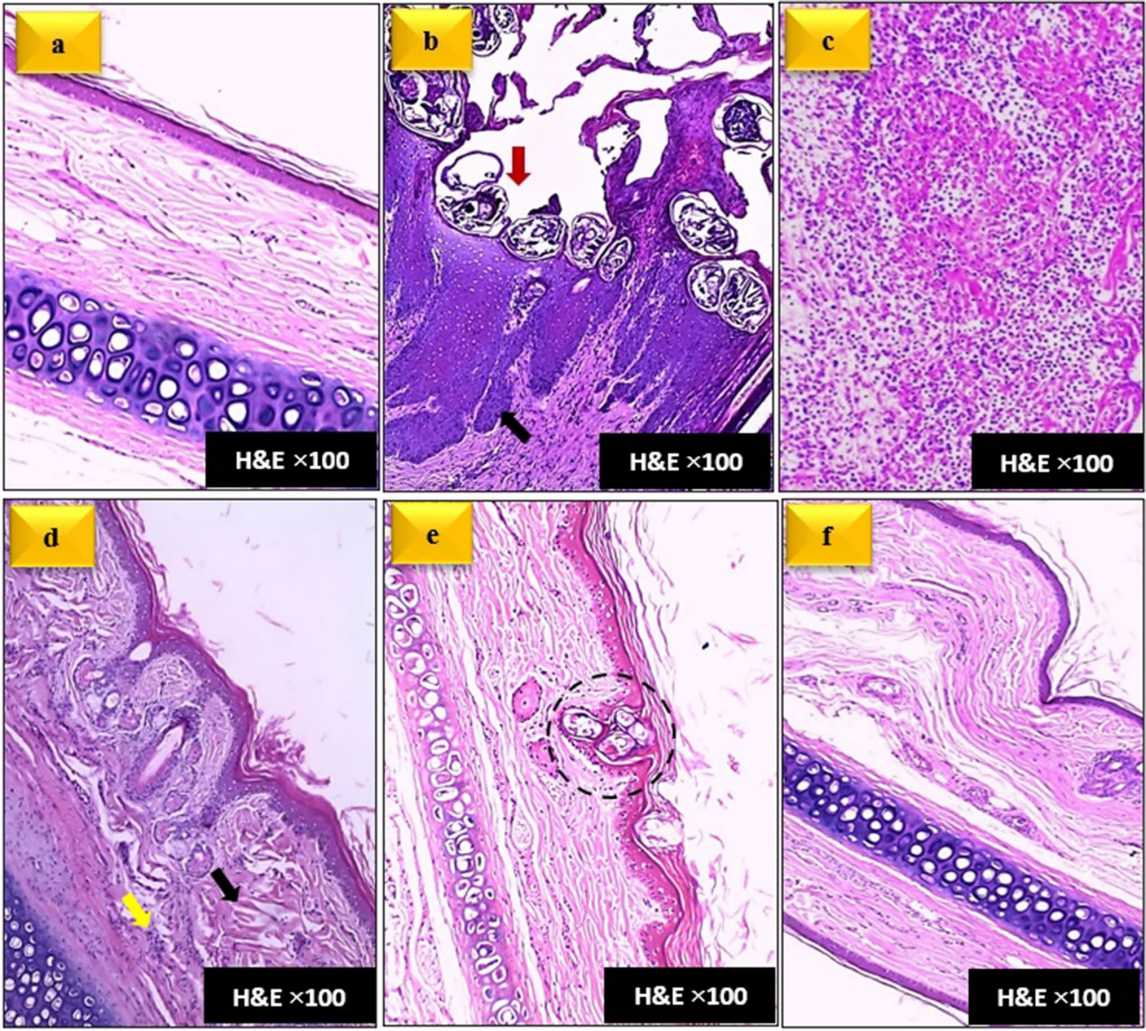
Photomicrographs of ear skin sections. Figure (a): normal ear skin section. Figures (b) and (c): skin sections from the untreated mangy rabbits, showing heavy mite infiltration "red arrow", rete ridges hypertrophy "black arrow" (b); and diffuse heavy dermal inflammatory infiltration (c). Figures (d) and (e): skin sections from the IVM-treated rabbits at D56 post-treatment, showing absence of motile mite stages, mild thickening of epidermis, mild dermal inflammatory infiltrate "yellow arrow", collagen deposits in dermis "black arrow" (d), with occasional trapping of eggs in epidermis "dashed circle" (e). Figure (f): skin section from FLR-treated group at D56 post-treatment, showing non infested epidermis with normal epidermal architecture and few inflammatory perivascular infiltrates in dermis (stain and magnification is mentioned on each figure).

Skin sections from the positive control group showed heavy crusts honeycombed with burrows containing different stages of *S. scabiei* mites. The epidermis exhibited marked acanthosis with pseudo-epitheliomatous hyperplasia (characterized by irregular rete pegs hypertrophy), epidermal microabscesses, and mild to moderate spongiosis. The dermis showed diffuse heavy leukocytic infiltrates, mainly with neutrophils, eosinophils, lymphocytes, plasma cells, and to a lesser extent mast cells. The intensity of the dermal inflammatory reactions seemed to be correlated with the intensity of epidermal lesions. Sebaceous glands were generally hyperplastic (Fig. 7_b,c_).

After 56 days of treatment with IVM, microscopic findings of treated rabbits showed improvement in skin lesions evidenced with healing of most of skin lesions and absence of mobile mite stages (adults, nymphs, and larvae). However, occasional trapping of some eggs in cornified epithelium was noted. Additionally, epidermal and dermal resolution was not complete when compared with skin sections obtained from healthy rabbits, as evidenced by mild epidermal thickening and mild inflammatory cell infiltrate in the superficial dermis, mainly lymphocytes, plasma cells, and to less extent neutrophils. Dermal collagen deposits were also noted (Fig. 7_d,e_). Regarding skin sections obtained at D56 post-treatment from rabbits treated with FLR, complete resolution of skin lesions and restoration of the normal skin architecture was noted (Fig. 7_f_). Results of the histopathological scoring were summarized in Table 5.

**Table 5:** Histopathological score in different groups

	Mean	±	S. D.	F. test	*P* value	Post Hock test			
Normal	0	±	0.00	89.476	0.001***	*P1*	0.001***	*P4*	0.001***
Control	20.4	±	0.89			*P2*	1.0*	*P5*	0.001***
IVM	4.40	±	1.14			*P3*	0.001***	*P6*	0.001***
FLR	0	±	0.00						

- *P1*: Normal & Control, *P2*: Normal & FLR, *P3*: Normal & IVM, *P4*: Control & FLR, *P5*: Control & IVM, *P6*: FLR & IVM

- Superscripts indicates statistical significance: ^(a)^ insignificant: *P* > 0.05, ^(*)^ Significant: *P* < 0.05, ^(**)^ very significant: *P*<0.01, ^(***)^ highly significant: *P* <0.001

- *n*= 10 in all groups except normal group, where *n*=5

### Biochemical evaluation

**Fig. 8:**
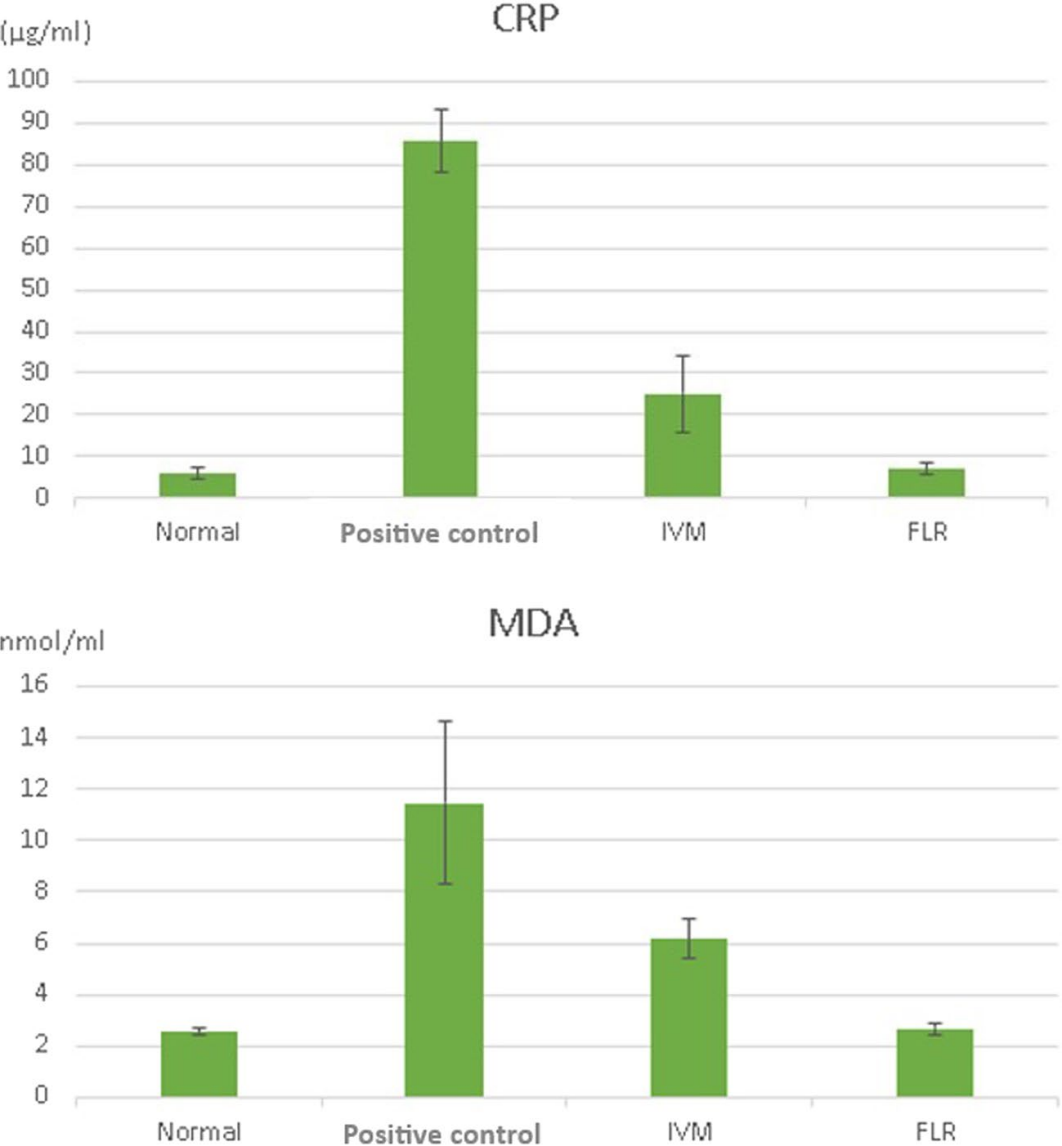
Serum levels of CRP and MDA (mean + SD).

The positive control group showed a highly significant increase in serum levels of CRP and MDA (*P*= 0.001***). At D56 post-treatment, the IVM-treated group showed a highly significant improvement in both parameters when compared with the positive control group (*P*= 0.001***). However, a highly significant increase in serum levels of both parameters was still present when compared with the negative control group. No statistically significant differences were recorded in the FLR-treated group regarding these parameters, when compared with the negative control group (*P*= 0.796 for CRP and 0.907 for MDA) (Fig. 8).

## Discussion

Since the *Sarcoptes* mite has an average 14-day life cycle and few acaricides have ovicidal effects, it is unlikely that single-dosage acaricides will completely clear infestation unless it could be retained in the host at therapeutically active concentrations levels to kill any newly hatched eggs throughout the mite's lifecycle. Therefore, development of acaricides with ovicidal activity is considered a research priority (Bernigaud et al. 2020). Additionally, considering the host-specific pharmacokinetics of acaricides is critical (Takano et al. 2023). Currently, the new family of isoxazolines is under investigation for scabies treatment. In our study, both *in vitro* and *in vivo* assessment of FLR were done in comparison with IVM (the only approved oral drug for cases of CS in humans).

Our *in vitro* study revealed that FLR was superior to IVM in terms of acaricidal activity against scabies mites when both drugs were applied in the same concentrations (50, 100 μg/ml). Lamassiaude et al. (2021) reported that *in vitro* application of 100 μM lotilaner, another isoxazoline drug, to human lice led to death of all lice 3 hours after exposure, whereas death rate reached 30 % only 3 hours after application of the same concentration of IVM. Interestingly, Mounsey et al. (2017) reported that the median *in vitro* survival times with IVM were doubled for scabies mites after 10 years of IVM use. Such finding could partly explain the delay in mites' death in our study.

Our study revealed that a single dose of FLR resulted in a better clinical improvement with no apparent side effects, when compared with the repeated weekly doses of IVM. Complete clinical recovery was noticed in the FLR-treated group in our study by D28 post-treatment. Interestingly, Wilkinson et al. (2021) reported that clinical recovery was noticed in mangy wombats treated with FLR as early as 21 days post-treatment. The IVM-treated group in our study showed complete clinical recovery by D35 post-treatment. Interestingly, Lee-Chow and Eshar (2020) reported that no clinical improvement was noticed in a mangy miniature pig after receiving two doses of IVM (300 μg/kg body weight) given subcutaneously at 10-days interval.

The current work revealed no apparent side effects in the FLR-treated animals. Similarly, Hyun et al. (2019) reported that no side effects were noticed following FLR administration, apart from a single bout of diarrhea that was observed in one raccoon dog that received a higher dose of FLR (52.3 mg/kg body weight). Interestingly, FLR was also demonstrated to be safe and well-tolerated in a preliminary study in wombats using a high dosage of 85 mg/kg (Wilkinson et al. 2021).

Regarding the adverse effects related to IVM in our study, diarrhea and decreased food intake were noticed, especially during the first few days following each dose of IVM. Abu Hafsa et al. (2021) reported that rabbits treated with IVM that was given in a dose of 200 μg/kg body weight twice weekly suffered from lower body weight and inappetence. The authors attributed this to oxidative stress caused by IVM therapy and supported this hypothesis by the progressive improvement in appetite and body weight gain in groups that received turmeric extract.

Conversely, Kaplaywar et al. (2017) reported that no side effects were encountered in rabbits treated with IVM that was given in three doses of 200 μg/kg body weight, with weekly interval in between. The discrepancy between such report and results encountered in our study may be related to the higher dose used in our work. Also, the poor general condition of the rabbits treated with IVM in the current study may be an additional factor that made them more susceptible to the side effects of IVM therapy.

While the FLR-treated group in our study showed parasitological cure starting from D10 post-treatment, Singh et al. (2022) reported that parasitological cure was delayed till D45 post-treatment. Interestingly, Hyun et al. (2019) reported that parasitological cure was noticed as early as D7 post-treatment in FLR-treated mangy raccoon dogs. Negative skin scrapings were noticed in our study in the IVR-treated group starting from D14 post-treatment. Similar findings were reported by Kaplaywar et al. (2017) in a study performed on mangy New Zealand rabbits treated with three doses of IVM (200 μg/kg body weight), with weekly interval in between. Mohamed et al. (2017) reported parasitological cure starting from D10 post-treatment with IVM therapy.

This discrepancy in timing of the clinical cure and the parasitological cure between different studies may be related to differences in the infection intensities, optimal dose delivery, the timing of treatment, the nature of the host, and the host's immune status. Difference in the environmental sanitary measures during treatment is another important factor. Notably, the time gap noticed between the parasitological cure and the clinical cure in both treated groups in our study is possibly due to dead mites and mites' antigens remaining in the host's skin, leading to local irritation. It takes some time after successful treatment for skin lesions to resolve completely.

Our study revealed that almost all eggs collected from the three groups were able to hatch in the incubator, suggesting that both IVM and FLR had no ovicidal activity. Our findings can be explained by the neurologically immature, nonmotile eggs not being susceptible to these neuroinhibitors. Similarly, Bernigaud et al. (2018) reported that afoxolaner, another isoxazoline drug, had no ovicidal effect on scabies mites, and Lamassiaude et al. (2021) reported that both IVR and lotilaner had no effect on nits of the human body lice. Despite being non-ovicidal, FLR have longer half-life than IVR, potentially making it more effective (Miglianico et al. 2018). This is particularly advantageous in treating CS, in which the administration of drugs that require multiple dosing could be challenging.

Poor compliance with stringent repeat-treatment regimens when applying non-ovicidal medications is a critical issue in CS control and is likely a major cause of treatment failure, highlighting the unmet need for innovative treatments that target the mite's eggs as well as the motile stages. Interestingly, Feng et al. (2023) noticed that molting *Sarcoptes* mites are less vulnerable to IVM than active mites. As a result of hatching eggs and mite resistance during the molting process, mites may survive despite two doses of IVM given 7 days apart.

Our histopathological findings regarding the skin sections from the infected untreated group were partly consistent with other previous reports in mangy rabbits (Abdelaziz et al. 2022) and other animal species like pigs (Debnath et al. 2020), dogs (Nwufoh et al. 2020b), Iberian ibex (Valldeperes et al. 2021), and wild boars (Sannö et al. 2021). Despite this consistency, differences in skin lesions were seen, even in animals of the same species, presumably due to differences in genetic background, immune responses, stage of the disease, infection intensities, and presence or absence of secondary infections in various hosts (Nimmervoll et al. 2013).

The current study revealed that resolution of the pathological changes in skin sections was superior in the FLR-treated group. The pathological changes that were noticed in skin sections from the IVM-treated group in our study were similar to those reported by Metwally (2017). Generally, the local histopathological findings reported in skin sections from untreated and treated mangy rabbits in our study are in consistency with the clinical and parasitological findings in the same study.

The state of systemic inflammation and oxidative stress noticed in the positive control group in our study could be mediated by mites-induced release of proinflammatory cytokines, since mites' antigens could upregulate the secretion of proinflammatory cytokines from keratinocytes, fibroblasts, and endothelial cells, thus promoting inflammation (Nwufoh et al. 2020a). Our results were in accordance with results reported by De et al. (2020) in a study performed on Nicobari pigs with CS. The magnitude of increase in serum acute phase proteins (APPs) is generally related to the severity of infection and extent of tissue damage. Meanwhile, different types of triggers such as parasitic, bacterial, or traumatic could stimulate different APPs.

Interestingly, the FLR-treated group in our study exhibited comparable results of both serum CRP and MDA to those of the normal control with no significant difference, indicating complete resolution of the mites-induced state of systemic inflammation and oxidative stress. This finding was consistent with the reported clinical and histopathological findings in the FLR-treated group in the same study. Similarly, these findings were consistent with the report of the European Medicines Agency (2017), which confirmed that FLR is a safe drug when given in a single dose in rats.

Although there was a significant improvement in such parameters in the IVM-treated group in the current study, the mean values of serum CRP and MDA were still higher than those of the normal control group, indicating a degree of oxidative stress. Abdel-Rahman and Ali (2021) reported that IVM-treated animals showed a state of oxidative stress for 3 months post-treatment, while Abu Hafsa et al. (2021) reported that use of antioxidants in concurrent with IVM therapy could result in a faster clinical recovery during treatment of mangy animals. Oxidative stress reported in the IVM-treated group in our study could partly explain the delayed recovery of histopathological lesions in skin sections.

## Conclusions

Conclusively, FLR-treated group exhibited a better recovery without apparent side effects. It appears that FLR is a better choice in alleviating both local and systemic consequences of CS. Hence, it is a promising scabicidal agent that is recommended to be studied for possible human use in control programs.

## Data Availability

The authors confirm that the data supporting the findings in this study are available within the article and its supplementary material. Raw data that support the findings of this study are available from the corresponding author, upon reasonable request.

## Abbreviations

CRP: C-reactive protein
CS: crusted scabies
D: day
FLR: fluralaner
IVM: ivermectin
MDA: malondialdehyde
*S*: *scabiei*: *Sarcoptes scabiei*
